# Effect of the glucagon-like peptide-1 analogue liraglutide on coronary microvascular function in patients with type 2 diabetes – a randomized, single-blinded, cross-over pilot study

**DOI:** 10.1186/s12933-015-0206-3

**Published:** 2015-04-22

**Authors:** Rebekka Faber, Mette Zander, Adam Pena, Marie M Michelsen, Naja D Mygind, Eva Prescott

**Affiliations:** Department of Cardiology, Bispebjerg University Hospital, Bispebjerg Bakke 23, 2400 Copenhagen NV, Denmark; Department of Endocrinology, Bispebjerg University Hospital, Bispebjerg Bakke 23, 2400 Copenhagen NV, Denmark; Department of Cardiology, Gentofte University Hospital, Kildegårdsvej 28, 2900 Hellerup, Copenhagen, Denmark; Department of Cardiology, Rigshospitalet University Hospital, Blegdamsvej 9, 2100 Copenhagen Ø, Denmark

**Keywords:** Coronary microcirculation, Diabetes Mellitus, Type 2 diabetes, Glucagon-like peptide-1, Coronary flow reserve, Microvascular dysfunction, Endothelial function, Liraglutide, Intervention study, Cross-over design

## Abstract

**Background:**

Impaired coronary microcirculation is associated with a poor prognosis in patients with type 2 diabetes. In the absence of stenosis of major coronary arteries, coronary flow reserve (CFR) reflects coronary microcirculation. Studies have shown beneficial effects of glucagon-like peptide-1 (GLP-1) on the cardiovascular system. The aim of the study was to explore the short-term effect of GLP-1 treatment on coronary microcirculation estimated by CFR in patients with type 2 diabetes.

**Methods:**

Patients with type 2 diabetes and no history of coronary artery disease were treated with either the GLP-1 analogue liraglutide or received no treatment for 10 weeks, in a randomized, single-blinded, cross-over setup with a 2 weeks wash-out period. The effect of liraglutide on coronary microcirculation was evaluated using non-invasive trans-thoracic Doppler-flow echocardiography during dipyridamole induced stress. Peripheral microvascular endothelial function was assessed by Endo-PAT2000®. Interventions were compared by two-sample *t*-test after ensuring no carry over effect.

**Results:**

A total of 24 patients were included. Twenty patients completed the study (15 male; mean age 57 ± 9; mean BMI 33.1 ± 4.4, mean baseline CFR 2.35 ± 0.45). There was a small increase in CFR following liraglutide treatment (change 0.18, CI95% [-0.01; 0.36], p = 0.06) but no difference in effect in comparison with no treatment (difference between treatment allocation 0.16, CI95% [-0.08; 0.40], p = 0.18). Liraglutide significantly reduced glycated haemoglobin (HbA1c) (-10.1 mmol/mol CI95% [-13.9; -6.4], p = 0.01), systolic blood pressure (-10 mmHg CI95% [-17; -3], p = 0.01) and weight (-1.9 kg CI95% [-3.6; -0.2], p = 0.03) compared to no treatment. There was no effect on peripheral microvascular endothelial function after either intervention.

**Conclusions:**

In this short-term treatment study, 10 weeks of liraglutide treatment had no significant effect on neither coronary nor peripheral microvascular function in patients with type 2 diabetes. Further long-term studies, preferably in patients with more impaired microvascular function and using a higher dosage of GLP-1 analogues, are needed to confirm these findings.

**Trial registration:**

ClinicalTrials.gov: NCT01931982.

**Electronic supplementary material:**

The online version of this article (doi:10.1186/s12933-015-0206-3) contains supplementary material, which is available to authorized users.

## Introduction

Patients with type 2 diabetes often suffer from both macro- and microvascular disease with cardiovascular disease being the most important contributor to mortality. Myocardial microvascular dysfunction is caused by reduced vasodilator reserve of the small coronary arteries. In case of dysfunctioning microvessels, blood flow in the larger coronary vessels does not increase sufficiently to meet oxygen demand. In the absence of stenosis of major coronary arteries, coronary microcirculation is reflected by the coronary flow reserve (CFR). CFR is reduced in patients with hypertension, obesity and diabetes [1,2]. Reduced CFR has been proven a strong predictor of poor prognosis in patients with suspected coronary artery disease (CAD) [3-5]. Recently, studies have shown CFR to provide independent prognostic information on patients with type 2 diabetes and no history of CAD. Patients with a low CFR had a significantly higher event rate of cardiovascular endpoints compared to patients with higher CFR [6,7].

Glucagon-like-peptid-1 (GLP-1) analogues are a group of drugs used in the treatment of type 2 diabetes. GLP-1 is an endogenous incretin hormone with pleiotropic metabolic effects [8]. GLP-1 analogues stimulate insulin release in a glucose dependent manner and reduce glucagon levels and appetite leading to reduced glycaemia and weight loss. Data from both animal- and clinical studies suggest beneficial effects of GLP-1 on the cardiovascular system. GLP-1 receptors are found throughout the gastrointestinal tract as well as in cardiomyocytes, endothelium and the sinoatrial node [9,10]. GLP-1 has been shown to enhance glucose uptake in the myocardium of isolated rat hearts and in dog myocardium [11,12]. Other potential beneficial effects of GLP-1 shown in animal studies include increased vasodilatation and coronary flow and activation of cardio protective pathways in murine heart models [13,14]. These changes were associated with improved survival and reduced infarct size after experimentally induced myocardial infarction [14].

In humans, Nikolaidis et al. [15] were the first to show an improvement of left ventricular ejection fraction (LVEF) following infusion of GLP-1 in patients with acute myocardial infarction. Other studies have found improvement of LVEF in patients with CAD during a dobutamine stress test [16] and in patients with chronic heart failure [17]. The mechanism behind this improvement is unclear. However, one study has found an increase in global myocardial blood flow in eight patients with type 2 diabetes following 6 hours of GLP-1 analogue infusion [18]. Similarly, a study by Subaran et al. has shown GLP-1 infusions recruit cardiac muscle microvasculature and increase myocardial blood flow in healthy humans [19]. Recently, animal studies have found GLP-1 protects cardiac microvessels against oxidative stress and apoptosis in diabetic rats [20] and preserves microvascular myocardial function estimated by CFR in a swine model after experimentally induced cardiac arrest [21]. Therefore, we hypothesize that GLP-1 treatment can improve myocardial microvascular function assessed by CFR in patients with type 2 diabetes.

## Methods

**Figure 1:**
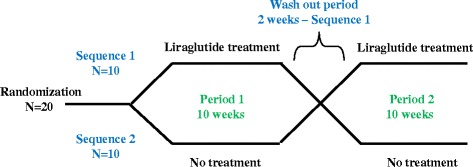
Study flow chart.

The study was a randomized single-blinded cross-over trial with a 1:1 allocation ratio. This design was chosen to allow each patient to serve as his or her own control. Figure 1 shows the study flow chart. Participants were randomized to receive the same treatment in different sequences either 1) 10 weeks of GLP-1 analogue followed by a 2 weeks wash-out period followed by 10 weeks of usual anti-diabetic therapy only (sequence 1) or 2) 10 weeks of usual anti-diabetic therapy only followed by 10 weeks of GLP-1 analogue (sequence 2). Hence, patients randomized to sequence 1 participated in the study for 22 weeks and patients randomized to sequence 2 participated for 20 weeks. All endpoints were evaluated at baseline, after 10, 12 and 22 weeks for sequence 1 and at baseline and after 10 and 20 weeks for sequence 2. The study was approved by The Danish Research Ethics Committee (H-3-2012-177), The Danish Health and Medicines Authorities (2012-005013-38) and has been continuously monitored by the GCP-unit at Bispebjerg University Hospital, Denmark. The study is registered at www.clinicaltrials.gov, with the ClinicalTrials.gov Identifier: NCT01931982.

Inclusion criteria were: type 2 diabetes on monotherapy with metformin or sulfonylurea (SU) or a combination, age 25-75 years, BMI > 25 kg/m2, HbA1c 42-86 mmol/mol, ability to speak and understand Danish or English and the mental ability to follow and understand the study, as assessed by the investigator.

Main exclusion criteria were: current treatment with insulin, DPP-4 inhibitor or GLP-1 analogue, history of cardiovascular disease including documented significant stenosis of the left anterior descending artery (LAD), allergy towards the test drug liraglutide (Victoza®), tool medicine dipyridamole or rescue medicine teophylline, chronic or previous acute pancreatitis, inflammatory bowel disease or pregnancy. Patients with severe co-morbidity or limited life expectancy were also excluded from the study. If dipyridamole stress test at baseline showed signs of significant ischemia the patient was excluded from the study. Patients were recruited from three different outpatient clinics in the capital region of Denmark. All data collection and analysis was carried out at Bispebjerg University Hospital. After screening for eligibility, written informed consent was collected for all participants. Inclusion was consecutive until 20 patients had completed the study.

The intervention consisted of 10 weeks of subcutaneous treatment with the GLP-1 analogue liraglutide (Victoza®) 6 mg/ml. Liraglutide was add-on to usual treatment (metformin, SU or metformin and SU) unless blood glucose levels indicated discontinuation of SU due to risk of hypoglycaemia. Patients were started on an initial dose of 0.6 mg once daily for two weeks to assess tolerability of the drug and to minimize potential gastrointestinal side-effects. After two weeks the dose was increased to 1.2 mg s.c once daily. The withdrawal criteria were: a) Patient’s wish to discontinue, b) Sustained gastrointestinal side-effects making the patients unable to increase liraglutide to full dose, and c) Repeated non-adherence to taking liraglutide as assessed by the investigator. Compliance was evaluated after one and two weeks of treatment by phone interviews. At the second compliance check, patients were also instructed to increase dosage to the full dose. Empty pens were collected from each patient after completion of the intervention to further assess compliance.

Primary endpoint was CFR assessed by a non-invasive trans-thoracic Doppler flow echocardiography. Secondary endpoint was peripheral endothelial function, assessed by Endo-PAT2000®. Additional endpoints were weight, waist circumference, blood pressure, heart rate, HbA1c and fasting C-peptide, plasma glucose and serum insulin for the calculation of Homeostatic Model Assessment (HOMA) index.

Calculation of sample size was based on the assumption that an improvement of 0.3 in CFR (i.e. approx. 15% of an expected mean CFR of 2) was regarded as clinically relevant. With an expected standard deviation within subject measurement of CFR of 0.3, alpha of 0.05 and statistical power of 0.9, we needed to study 13 patients in a cross-over design. However, considering the risk of drop-out we chose to include 20 patients.

### Coronary flow reserve

**Figure 2:**
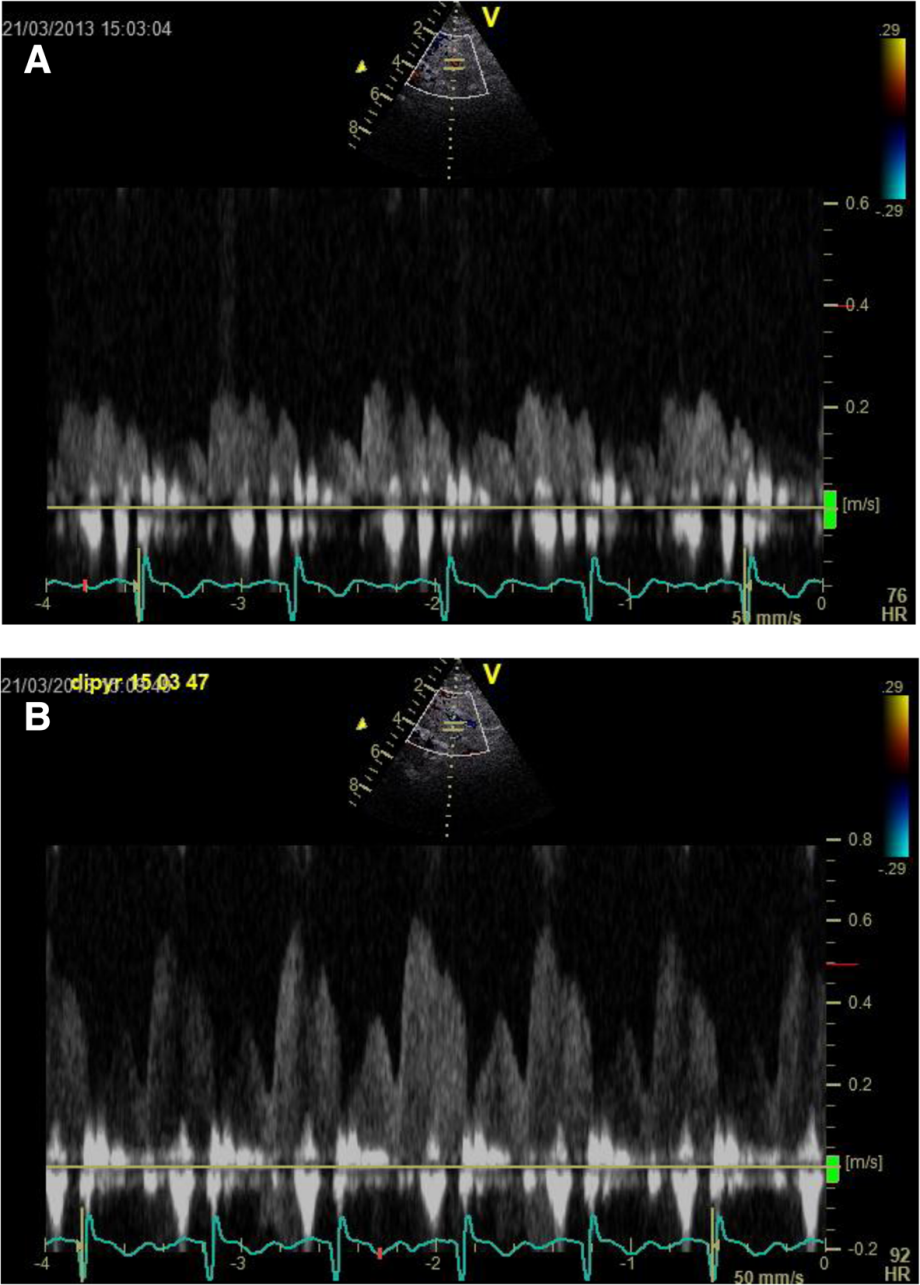
Measuring CFR by Doppler flow echocardiography. Left anterior descending artery (LAD) flow during rest **(A)** and LAD flow during dipyridamole induced stress **(B)**.

CFR was assessed non-invasively by trans-thoracic Doppler flow echocardiography. All patients were instructed to abstain from food and drinks containing xanthine (coffee, tea, cola, chocolate, and banana) for 24 hours before the examination. Use of nitroglycerin and antihypertensive drugs were also discontinued for 24 hours. With the patient in the left supine position, the LAD was identified in the best possible view. Whether the location was at the proximal, mid-distal or distal part of the artery depended on patient variability such as respiration pattern and degree of obesity. After locating the artery the ultrasound probe was rotated and tilted to obtain the best possible Doppler signal. Coronary flow velocity was measured at a minimum of 3 cardiac circles at rest and during peak pharmacological stress induced by dipyridamole infusion (0.84 mg/kg) for 6 minutes. Patients were monitored with blood pressure and 3-lead electrocardiogram before and during the infusion. Care was taken to maintain the position and angle of the probe. Immediately after the examination, peak velocities at stress and at rest were measured and CFR was calculated as the ratio of flow during stress to during rest, see Figure 2. After the examination, the patient was observed for 30 minutes before leaving the site. The investigator was blinded to which group the patient belonged to throughout the study.

### Endothelial function

Peripheral microvascular endothelial function was assessed with the commercially available Endo-PAT2000® (Itamar Medical, Caesarea, Israel). This is a non-invasive, observer-independent technique that offers automatic data-analysis [22]. Endo-PAT2000® uses peripheral arterial tonometry (PAT) technology to measure volume changes in the fingertip before and after occlusion. Measurements from the contra-lateral arm are used to control for non-endothelial dependent changes in vascular tone. The automatically calculated ratio (reactive hyperaemia index = RHI) provides an index for endothelial function.

Blood pressure and heart rate were recorded three times with 2 minutes interval before the examination. The mean of the last two recordings served as baseline blood pressure and heart rate. The examination was performed in a fasting state in a quiet dark room with room temperature 21-24°C using bilateral digital probes. A baseline recording was performed for a minimum of 6 minutes followed by 5 minutes of cuff occlusion of the non-dominant arm. Occlusion was aimed at a minimum of 60 mmHg above the baseline systolic blood pressure to ensure complete occlusion. Cuff was released and digital flow was recorded in 5 minutes and the relative increase in flow adjusted for the non-tested arm was automatically calculated (RHI).

During the examination, several unintended actions can interrupt the PAT signal. This includes noisy signal, sudden movement of one arm if the patient falls asleep and incomplete occlusion of the cuff (the pressure falls under 60 mmHg above the systolic pressure at baseline). The machine will then add a warning signal to the automatically calculated RHI index.

### Additional secondary outcomes

Blood samples were taken in a fasting state. Samples for HbA1c were analysed using standard methods. Samples for plasma glucose, serum insulin and serum C-peptide were taken twice with an interval of 15 minutes and immediately put on ice. An average of the two samples was used for later analysis. Plasma glucose was measured using YSI 2300 STAT Plus Glucose Analyzer (YSI Life Sciences, Ohio, USA). Serum insulin and C-peptide were measured using AutoDELFIA® immunoassay systems (PerkinElmer, Massachusetts, USA). HOMA IR index was calculated as (fasting insulin (μU/ml) × fasting glucose (mmol/L))/22.5. Blood pressure recordings were measured as stated above in connection with the EndoPAT2000® examination. Subjects were defined as hypertensive if they were taking anti-hypertensive drugs or if blood pressure was above 140/90 mmHg. Dyslipidaemia was defined as triglyceride or cholesterol levels above normal range or patients on lipid lowering agent.

### Randomization procedure

Randomization concerning sequence of intervention was supervised by the regional GCP unit. The patient was asked to draw a number from a box containing 20 numbers. Numbers 1-10 represented sequence 1 and numbers 11-20 represented sequence 2. If a patient withdrew from the study, making inclusion of another patient necessary, the corresponding number was put back in the box. After randomization the patient was identified by a number and followed the study according to the sequence he or she was assigned to. A research assistant performed the randomization procedure and allocation. The investigators who performed the primary outcome examination by echocardiography had no knowledge of which sequence the patient belonged to.

### Statistical analyses

Carry over effects were measured using the pkcross command in Stata 13.1 for cross-over design experiment. The command calculates carry over, period, sequence and treatment effect. Using different parameterizations, the treatment effect can be measured in assumption of no carry over, period or sequence effect. Data was analyzed as two-sample *t*-test comparing changes within and between liraglutide treatment and no treatment allocations after ensuring there was no carry over, sequence or period effect. Paired two-sample *t*-test was used for within allocation comparisons whereas unpaired two-sample *t*-test was used for between treatment allocation comparisons. Continuous variables are expressed as mean ± SD, categorical variables as frequency and percentage. A p-value of ≤ 0.05 was considered statistically significant. All analyses were performed in Stata 13.1 (Stata Statistical Software: Release 13. College Station, Texas, USA).

## Results

### Study population

**Figure 3:**
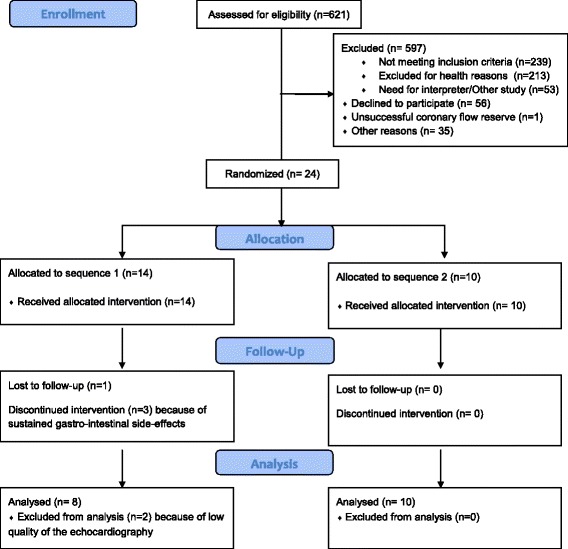
Participant flow chart.

We assessed 621 patients who were followed for type 2 diabetes in outpatient clinics for eligibility (Figure 3). 213 patients were excluded due to co-morbidity, primarily previous CAD (37%, including AMI, PCI and CABG), reduced renal function (13%) and gastrointestinal disease (12%, including inflammatory bowel disease, previous pancreatitis and gastric bypass). Eligible patients (n = 81) were invited to participate. Of these, 56 declined. The main reason for reluctance to participating in the study was the concern for potential side-effects to the test drug liraglutide (Victoza®). Twenty-four patients were randomized, 14 to sequence 1 and 10 to sequence 2. During the follow up period from 15.05.2013 to 15.04.2014 one patient was lost to follow up and three patients withdrew due to sustained gastrointestinal side-effects to the liraglutide treatment in sequence 1. There were no withdrawals in sequence 2. Liraglutide was generally well tolerated and side-effects were transient with duration of approximately two weeks. Background therapy needed readjustment during liraglutide treatment in 3 out of the 4 patients receiving both metformin and SU. In one case, SU dosage was halved and in two cases SU was paused during liraglutide treatment due to HbA1c levels indicating risk of hypoglycaemia. No incidents of hypoglycaemia were observed. Three patients developed angina-like symptoms and were referred to either stress echocardiography or CT angiography to rule out obstructive CAD. None of these tests came out positive and the patients continued the study protocol. In both sequences, a total of 10 patients received the full 10 weeks liraglutide treatment.

Table 1 describes baseline characteristics and echocardiographic data of the two sequences and the total study population. Participants were predominantly male (75%), with a mean age of 57 ± 9 years, were obese (BMI 33.1 ± 4.4) and had a mean HbA1c of 52.8 ± 8.2 mmol/mol. There were a high percentage of known cardiac risk factors such as hypertension (80%) and dyslipidaemia (95%) in the study population. Echocardiographic data showed a LVEF within the normal range (mean 54.6 ± 5.8). CFR ranged from 1.69 to 3.11 (mean 2.35 ± 0.45).

**Table 1:** **Baseline characteristics and echocardiographic data of study subjects**

**Variables**	**Sequence 1 (N = 10)**	**Sequence 2 (N = 10)**	p-value*	**Total study population (N = 20)**
Age (years)	57 ± (9)	56 ± (9)	0.87	57 ± (9)
Male gender	7 (70%)	8 (80%)	0.63	15 (75%)
Duration type 2 diabetes (years)	4 ± (3)	5 ± (3)	0.35	4 ± (3)
BMI (kg/m2)	34.8 ± (4.1)	31.4 ± (4.1)	0.08	33.1 ± (4.4)
Waist circumference (cm)	119 ± (11)	111 ± (8)	0.08	115 ± (10)
HbA1c (mmol/mol)	51.1 ± (4.8)	54.5 ± (10.6)	0.37	52.8 ± (8.2)
eGFR (ml/min/1.73 m2)	89 ± (3)	87 ± (6)	0.37	88.2 ± (4.8)
Haemoglobin (mmol/L)	9.0 ± (0.5)	8.9 ± (0.7)	0.75	8.9 ± (0.6)
Hypertension	7 (70%)	9 (90%)	0.29	16 (80%)
Dyslipidaemia	10 (100%)	9 (90%)	0.33	19 (95%)
Micro albuminuria**	0 (0%)	3 (30%)	0.07	3 (15%)
Diabetes treatment:				
Biguanide	10 (100%)	10 (100%)	1.00	20 (100%)
Sulfonylurea	1 (10%)	3 (30%)	0.29	4 (20%)
Other treatment:				
Statin	8 (80%)	7 (70%)	0.63	15 (75%)
ACE inhibitor	2 (20%)	4 (40%)	0.36	6 (30%)
Beta blocker	0 (0%)	3 (30%)	0.07	3 (15%)
LVEF (%)	52.8 ± (5.4)	56.3 ± (6.0)	0.17	54.6 ± (5.8)
Peak diastolic flow at rest (cm/sec)	0.26 ± (0.06)	0.26 ± (0.06)	0.94	0.26 ± (0.06)
Peak diastolic flow during stress (cm/sec)	0.58 ± (0.12)	0.64 ± (0.17)	0.35	0.61 ± (0.14)
CFR	2.26 ± (0.48)	2.44 ± (0.44)	0.39	2.35 ± (0.45)

Data are presented as mean (± SD) or n (%). *P-value for difference in variables between sequence 1 and sequence 2. Two-sided T-tests were used. **Micro albuminuria was defined as (U-Albumin/U-Creatinine Ratio > 30). *Abbreviations*: *HbA1c* glycated haemoglobin, *eGFR* estimated glomerular filtration rate, *LVEF* Left ventricular ejection fraction, *CFR* coronary flow reserve.

### Coronary flow reserve

In two of the 70 echocardiographies performed the investigator was unable to identify LAD in the same segment compared to previous examinations for the same patient and recordings obtained at these two examinations were of poor quality. Thus, 2 patients were excluded from CFR assessment in sequence 1 because of unreliable comparability with previous examinations. Analysis of CFR was then carried out for 8 patients in sequence 1 and 10 patients in sequence 2. Adverse reactions to dipyridamole included flushing, headache, shortness of breath and increased heart rate, but generally dipyridamole was well tolerated. No cardiac arrhythmias were observed during the dipyridamole infusions.

Table 2 shows changes in CFR values before and after no treatment and liraglutide treatment allocation. There was no significant change in CFR during no treatment (before 2.45 ± 0.41; after 2.47 ± 0.42, p = 0.82). Liraglutide treatment caused a small increase in CFR, which was of borderline significance (before 2.25 ± 0.31; after 2.43 ± 0.39, p = 0.06).

**Table 2:** **Mean (SD) for CFR and secondary outcomes before and after treatment allocations**

**Variable**	**No treatment value before**	**No treatment value after**	p-value*	**Liraglutide value before**	**Liraglutide value after**	p-value*
CFR	2.45 (0.41)	2.47 (0.42)	0.82	2.25 (0.31)	2.43 (0.39)	0.06
RHI	1.84 (0.41)	1.87 (0.35)	0.70	1.89 (0.47)	2.00 (0.40)	0.31
AI	11.1 (15.4)	9.7 (16.5)	0.49	10.6 (17.9)	9.4 (14.0)	0.58
HbA1c (mmol/mol)	49.8 (9.3)	50.8 (8.9)	0.52	52.1 (7.2)	42.9 (4.0)	<0.0001
Weight (kg)	100.6 (10.1)	100.5 (10.4)	0.89	100.9 (10.8)	98.8 (11.7)	<0.01
Systolic BP (mmHg)	140 (12)	144 (14)	0.10	143 (14)	137 (12)	0.02
Diastolic BP (mmHg)	80 (6)	81 (9)	0.42	81 (7)	80 (6)	0.62
Fasting glucose (mmol/L)	7.74 (1.54)	8.26 (2.18)	0.23	8.44 (1.90)	6.83 (1.16)	<0.001
Insulin (pmol/L)	85 (46)	82 (41)	0.71	87 (45)	77 (41)	0.37
C-peptide (pmol/L)	969 (362)	990 (337)	0.74	1046 (374)	1098 (401)	0.41
HOMA index	4.9 (2.8)	4.9 (2.6)	0.93	5.5 (3.4)	4.0 (2.4)	0.06

Data are presented as mean (SD). *P-value for the difference between values before versus after no treatment or liraglutide treatment, respectively. Two-sided T-tests were used under the assumption of no carry over effect. *Abbreviations*: *CFR* coronary flow reserve, *RHI* reactive hyperaemia index, *AI* augmentation index, *HbA1c* glycated haemoglobin, *BP* blood pressure, *HOMA* Homeostatic Model Assessment.

Table 3 shows the difference between changes in CFR in the no treatment and the liraglutide treatment allocation. The difference between treatment allocations was not statistically significant (0.16, CI95% [-0.08; 0.40], p = 0.18).

**Table 3:** **Mean (SD) for changes in CFR and secondary outcomes between treatment allocations**

**Variable**	**Changes during no treatment (SD)**	**Changes during liraglutide (SD)**	**Difference between changes (95% confidence interval)**	p-value*
CFR	0.02 (0.32)	0.18 (0.38)	0.16 [-0.08; 0.40]	0.18
RHI	0.03 (0.30)	0.12 (0.51)	0.09 [-0.17; 0.36]	0.49
AI	-1.4 (8.9)	-1.2 (9.6)	0.20 [-5.7; 6.1]	0.95
HbA1c (mmol/mol)	0.9 (6.3)	-9.2 (5.1)	-10.1 [-13.9; -6.4]	0.01
Weight (Kg)	-0.1 (2.5)	- 2.0 (2.8)	-1.9 [-3.6; -0.2]	0.03
Waist circumference (cm)	-1 (4)	-1 (2)	1 [-2; 2]	0.96
Systolic BP (mmHg)	5 (12)	-5 (9)	-10 [-17; -3]	0.01
Diastolic BP (mmHg)	1 (7)	-1 (8)	-2 [-7; 3]	0.35
Heart rate (bpm)	4 (10)	4 (9)	0.05 [-6; 6]	0.97
Fasting glucose (mmol/L)	0.52 (1.85)	-1.61 (1.54)	-2.13 [-3.24; -1.03]	<0.001
Insulin (μU/ml)	-3 (39)	-10 (45)	-6 [-34; 21]	0.65
C-peptide (pmol/L)	21 (280)	52 (279)	31 [-150; 212]	0.73
HOMA index	0.1 (3.6)	-1.5 (3.2)	-1.6 [-3.7; 0.5]	0.13

Data are presented as mean (SD). *P-value for the difference between changes in treatment allocations: liraglutide treatment versus no treatment. Two-sided t-tests were used under the assumption of no carry over effect. *Abbreviations*: *CFR* coronary flow reserve, *RHI* reactive hyperaemia index, *AI* augmentation index, *HbA1c* glycated haemoglobin, *BP* blood pressure, *HOMA* Homeostatic Model Assessment.

### Peripheral endothelial function

Analyses were carried out for all 20 patients. There were no changes in RHI in the no treatment allocation (before 1.84 ± 0.41; after 1.87 ± 0.35, p = 0.70) or the liraglutide treatment allocation (before 1.89 ± 0.47; after 2.00 ± 0.40, p = 0.31) and no between treatment allocation difference (0.09, CI95% [-0.17; 0.36], p = 0.49) (Table 3). Furthermore, there was no effect on arterial stiffness, measured by augmentation index (AI), following liraglutide treatment compared to no treatment (0.20, CI95% [-5.7; 6.1], p = 0.95). Results were similar when examinations with a warning signal were removed (n = 6) (difference between changes in RHI in the two treatment allocations (0.19, CI95% [-0.07; 0.45], p = 0.14).

### Additional secondary outcomes

Liraglutide treatment was associated with a significant decrease in HbA1c compared with no treatment (-10.1 mmol/mol, CI95% [-13.9; -6.4], p = 0.01), a decrease in fasting plasma glucose (-2.13 mmol/L, CI95% [-3.24; -1.03], p < 0.001), a moderate weight loss (-1.9 kg, CI95% [-3.6; -0.2], p = 0.03) and a reduction in systolic blood pressure (-10 mmHg, CI95% [-17; -3], p = 0.01). Carry over effect was found for one variable, HbA1c, but taking this into the parameterization in the pkcross command, the effect of liraglutide remained significant (p = 0.01 versus p = <0.001). Please see Additional file 1 for carry over, period and sequence effect for all outcomes. There was no effect of liraglutide treatment on diastolic blood pressure (-2 mmHg, CI95% [-7; 3], p = 0.35), heart rate (0.05 bpm, CI95% [-6; 6], p = 0.97) or waist circumference (1 cm, CI95% [-2; 2], p = 0.96) compared with no treatment. Additionally, liraglutide had no effect on serum insulin (-6 μU/ml, CI95% [-34; 21], p = 0.65), serum C-peptide (31 pmol/L, CI95% [-150; 212], p = 0.73) or HOMA index (-1.6, CI95% [-3.7; 0.5], p = 0.13).

## Discussion

In the present study, we compared the changes in coronary flow reserve after 10 weeks of treatment with 1.2 mg liraglutide daily versus no treatment in patients with type 2 diabetes. Although liraglutide was associated with weight loss, lower systolic blood pressure and improved HbA1c, there was only a small and statistically insignificant improvement in CFR.

To our knowledge, apart from one short-acting GLP-1 infusion intervention study in healthy humans [19] no clinical trial has evaluated the effect of GLP-1 on coronary microcirculation. Few animal studies, including a rat and two swine models, have addressed coronary microcirculation following GLP-1 treatment. These studies found GLP-1 had a protective anti-oxidative effect on coronary microvessels [20,21,23]. Furthermore, Dokken et al. found GLP-1 preserved, but did not improve, myocardial microvascular function estimated by CFR in an acute setting of swine subjected to experimentally induced cardiac arrest. These effects were not associated with an improvement in survival or LVEF [23]. In the present study, the population consists of patients with type 2 diabetes and no history of CAD, myocardial ischemia or heart failure. In the previous clinical studies showing GLP-1 had a positive effect on the cardiovascular system the study populations suffered from acute myocardial infarction, CAD or chronic heart failure [15-17]. The positive effects found by others might be due to improved myocardial glucose uptake under myocardial ischaemic conditions [24] rather than direct effects on the cardiovascular system.

We did find a trend towards an increase in CFR in the liraglutide treatment allocation alone, although this increase was modest and only of borderline significance. It is possible that a short-term treatment study is inadequate to induce the structural changes needed for an improvement in the coronary microcirculation. Moreover, we cannot rule out that 1.8 mg or 3 mg (recently approved for weight loss) could have improved the effect of liraglutide on CFR further. However, we chose 1.2 mg as this dose is well accepted for glycaemic control and to minimize the risk of gastrointestinal side-effects.

The enrolled patients had a better coronary microvascular function than expected which may have limited the treatment effect (i.e., a ‘ceiling effect’). Furthermore, coronary microvascular dysfunction is known to be more common in women than in men [25] and some studies have shown higher incidents of microvascular dysfunction in African Americans compared to white Americans [26]. The current study population consists of Danish white Caucasians with a greater proportion of men (75%). GLP 1 treatment in women or patients with documented microvascular angina might have a different outcome.

Nevertheless, during liraglutide treatment CFR improved by 0.18 from a baseline value of 2.35 or equivalent of 8% and the upper limit of the confidence interval was 0.36 or 15%. Therefore, we are not likely to overlook clinically relevant improvements in this study population and in particular not improvements caused by direct effects of liraglutide. Diabetes, hypertension and overweight are strongly related to impaired CFR [1,2] and it is likely that any modest improvement in CFR is caused by the significant improvement in glycemic regulation, systolic blood pressure and moderate weight loss found in this study.

Our findings of liraglutide improving glycemic regulation, reducing systolic blood pressure and body weight are in concordance with several others studies [27-29]. On-going long-term clinical trials will clarify the cardiovascular effects of GLP-1 analogues [30]. Interestingly, the first studies designed to evaluate the long-term effects of DDP-4 inhibitors (enzyme responsible for the degradation of GLP-1) on the cardiovascular system came out neutral [31,32].

We found no effect of liraglutide treatment on peripheral endothelial function assessed by Endo-PAT2000®. Endo-PAT2000® has been validated against invasive measurements of endothelial function with good results [33] and has been linked to several traditional cardiovascular risk factors [22]. Furthermore, one study has evaluated its implication for clinical trials, and concluded that Endo-PAT2000® can detect treatment effects in relative small sample sizes (n = 15-30) [34]. However, the method differs from the ‘gold standard’ of assessing peripheral endothelial function by flow-mediated vasodilation and the validity of the RHI index derived as a measure of endothelial function has recently been questioned [35].

Our findings are in line with other studies that found no effect of GLP-1 analogues on peripheral endothelial function estimated by flow-mediated dilation in patients with type 2 diabetes and estimated by RHI in a pre-diabetic population [36,37]. In contrast, A. Gnasso and his group did find an improvement in endothelial function following GLP-1 analogue treatment [38] although this study was observational. A recent study by Rizzo et al. [39] found liraglutide significantly decreased carotid intima-media thickness, a marker of subclinical atherosclerosis, but whether this translates to positive influence on the peripheral microvascular endothelial function is unclear. Another clinical randomized trial found improvement of several markers of vascular function but failed to show an effect of GLP-1 on arterial stiffness (AI) which is in accordance with our findings [40]. Also, recent evidence suggests potential harmful effects of DDP4-inhibitors on peripheral endothelial function [41]. In summary, the current evidence on the effects of GLP-1 on peripheral endothelial function is conflicting and based on small studies [42]. Our findings contribute to a negative position towards a GLP-1 potential for improving peripheral endothelial function in patients with type 2 diabetes.

### Strengths and limitations

The applied technique of measuring CFR non-invasively with Doppler flow echocardiography is technically demanding. The technique is used routinely in our echolab and has been validated in previous studies against invasive measurements [43,44] and PET [45] with good agreement. Alternative methods for assessing coronary microcirculation are invasive by thermo dilution or intracoronary Doppler flow assessment or by PET CT. PET CT has less availability, is associated with radiation and there is no evidence to suggest that PET CT is a more sensitive measure than transthoracic Doppler flow echocardiography. Cardiac Magnetic Resonance Imaging (MRI) is a promising method that has not yet been standardized for quantitative assessment of coronary microvascular function [46]. We chose to use transthoracic Doppler echocardiography because it is non-invasive, reproducible and does not expose the patients repeatedly to radiation.

We found the standard deviation of the difference between two CFR values for the same patient to be higher than expected (0.38 versus 0.30). Although we did include a greater number of patients than the statistical power estimation required, the actual higher SD increases the risk of a type 2 error in this study. However, as discussed above, we are not likely to overlook clinically relevant improvements and in particular no improvements caused by direct effects of liraglutide.

The population consisted of patients with type 2 diabetes without symptoms or history of CAD by inclusion in the study. Three patients developed angina-like symptoms but had no signs of significant coronary lesions in additional examinations. We cannot rule out that study participants could develop asymptomatic CAD over the course of the study but this is unlikely given the limited duration.

This study was not placebo controlled because it was not sponsored by pharmaceutical companies and no placebo medication could be provided. Instead, patients received only background therapy during a control period, which was then compared to liraglutide treatment.

The size of the study population was small in this pilot study. The sample size of the study was determined by power calculations and the number of patients reduced by using the cross-over design. Calculations were based on the assumption that an improvement in CFR of 0.3 was clinically relevant. We found a difference in CFR between treatment allocations of 0.16 with a 95% CI of [-0.08; 0.40]. An improvement of 0.16 or more may be clinically relevant if confirmed in larger trials.

Finally, it is possible that the two weeks wash-out period was insufficient to avoid cardiovascular effects of liraglutide to be referred. However, this limitation was addressed by calculating carry over effect which was non-significant for the primary outcome CFR.

## Conclusion

Despite a significant weight-loss, reduction in HbA1c and systolic blood pressure, we found only a small and non-significant improvement in CFR after 10 weeks treatment with liraglutide. In our short-term treatment study, we therefore conclude that the GLP-1 analogue liraglutide does not have any significant effect on coronary microcirculation in patients with type 2 diabetes. We also conclude that liraglutide has no effect on peripheral endothelial function in patients with type 2 diabetes. This study contributes to the highly important clinical issue of improving cardiovascular risk profile in patients with type 2 diabetes. Further long-term studies, preferably in patients with more impaired microvascular function and using a higher dosage of GLP-1 analogues, are needed to confirm these findings.

## Additional file

Additional file 1:
**Carry over, period and sequence effect for primary and secondary outcomes after performing analysis of variance (ANOVA) for a cross-over study.** Data are presented as p-values for carry over, period and sequence effect. ANOVA-analysis for cross-over design experiments were used (pkcross command in STATA 13.1). Abbreviations: CFR coronary flow reserve, RHI reactive hyperaemia index, AI augmentation index, HbA1c glycated haemoglobin, BP blood pressure, HOMA Homeostatic Model Assessment.

## Abbreviations

CFR: Coronary flow reserve
GLP-1: Glucagon-like peptide-1
HbA1c: Glycated haemoglobin
CAD: Coronary artery disease
LVEF: Left ventricular ejection fraction
LAD: Left anterior descending artery
SU: Sulfonylurea
HOMA: Homeostatic Model Assessment
PAT: Peripheral arterial tonometry
RHI: Reactive hyperaemia index
MRI: Magnetic Resonance Imaging
