# Supplementary material for: Effect of the glucagon-like peptide-1 analogue liraglutide on coronary microvascular function in patients with type 2 diabetes – a randomized, single-blinded, cross-over pilot study
Source: Cardiovasc Diabetol. 2015 Apr 22;14:41. doi: 10.1186/s12933-015-0206-3 (PMC4407869; doi:10.1186/s12933-015-0206-3)
Supplement: Additional file 1: — Carry over, period and sequence effect for primary and secondary outcomes after performing analysis of variance (ANOVA) for a cross-over study. Data are presented as p-values for carry over, period and sequence effect. ANOVA-analysis for cross-over design experiments were used (pkcross command in STATA 13.1). Abbreviations: CFR coronary flow reserve, RHI reactive hyperaemia index, AI augmentation index, HbA1c glycated haemoglobin, BP blood pressure, HOMA Homeostatic Model Assessment. [file 12933_2015_206_MOESM1_ESM.pdf]

Additional file 1: Carry over, period and sequence effect for primary and secondary outcomes after performing analysis of variance (ANOVA) for a cross-over study.

Variable	Carry over effect (p-value)	Period effect (p-value)	Sequence effect (p-value)
CFR	0.85	0.51	0.83
RHI	0.73	0.09	0.74
AI	0.29	0.85	0.26
HbA1c (mmol/mol)	0.05	0.44	0.07
Weight (Kg)	0.20	0.74	0.29
Waist Circumference (cm)	0.12	0.49	0.18
Systolic BP (mmHg)	0.81	0.51	0.78
Diastolic BP (mmHg)	0.38	0.95	0.24
Heart rate (bpm)	0.68	0.27	0.54
Fasting glucose (mmol/L)	0.75	0.06	0.69
Insulin (µU/ml)	0.54	0.41	0.48
C-peptide (pmol/L)	0.71	0.95	0.60
HOMA index	0.57	0.77	0.43

Data are presented as p-values for carry over, period and sequence effect. ANOVA-analysis for cross-over design experiments were used (pkcross command in STATA 13.1).
Abbreviations: *CFR* coronary flow reserve, *RHI* reactive hyperaemia index, *AI* augmentation index, *HbA1c* glycated haemoglobin, *BP* blood pressure, *HOMA* Homeostatic Model Assessment.
